# MiR‐138 inhibits cell proliferation and reverses epithelial‐mesenchymal transition in non‐small cell lung cancer cells by targeting GIT1 and SEMA4C

**DOI:** 10.1111/jcmm.12666

**Published:** 2015-08-18

**Authors:** Jiefang Li, Qinrong Wang, Ruiling Wen, Jieman Liang, Xiaoling Zhong, Wei Yang, Dongxiang Su, Jun Tang

**Affiliations:** ^1^ KingMed Diagnostics and KingMed School of Laboratory MedicineGuangzhou Medical UniversityGuangzhouChina

**Keywords:** miRNA, non‐small‐cell lung cancer, proliferation, EMT, GIT1, SEMA4C

## Abstract

Non‐small‐cell lung cancer (NSCLC) is one of the most common and lethal malignant tumours worldwide with a poor 5‐year survival rate. Recent studies indicated that miRNAs have been involved in the tumorigenic driver pathways in NSCLC, but the relevant molecular mechanisms are not well‐understood. In this study, we investigated the biological functions and molecular mechanisms of miR‐138 in human NSCLC. The effects of miR‐138 on the NSCLC cell growth and epithelial‐mesenchymal transition (EMT) were first examined. Then the targeting connections of miR‐138 with G‐protein‐coupled receptor kinase‐interacting protein 1 (GIT1) and semaphorin 4C (SEMA4C) were confirmed by dual luciferase reporter assays. Finally, the effects of GIT1 and SEMA4C on the NSCLC cell growth and EMT were investigated respectively. We found that the ectopic expression of miR‐138 resulted in a significant inhibition of NSCLC growth and reversion of EMT. GIT1 and SEMA4C were identified as two novel targets of miR‐138. Furthermore, GIT1 and SEMA4C knockdown inhibited the cell growth and reversed EMT, just like the effects of miR‐138 overexpression on NSCLC cells, whereas ectopic expression of GIT1 and SEMA4C partly rescued the suppressive effects of miR‐138 in NSCLC cells. These data represent a crucial step towards the understanding of the novel roles and molecular mechanism of miR‐138, GIT1 and SEMA4C in NSCLC progression, which may provide some new targets or prognostic biomarkers for NSCLC treatment, thus having implications in translational oncology.

## Introduction

Non‐small‐cell lung cancer (NSCLC) is one of the most common and lethal malignant tumours worldwide and accounts for about 80% of the total lung cancer cases 1, 2, 3. Despite improvements in clinical diagnosis and therapeutic strategies, the 5‐year survival rate for NSCLC still remains between 10% and 20% 1, 2, 3, 4, 5, 6. To provide new insight that will facilitate the development of new diagnosis and therapeutic strategies, it is crucial to understand the molecular mechanisms that promote the development and progression of NSCLC cells.

Cell proliferation and epithelial‐mesenchymal transition (EMT) are two of the most important malignant characteristics in NSCLC cells 7, 8. During EMT, the morphology of epithelial cells will transform to a mesenchymal appearance; meanwhile, the epithelial cells would adopt some mesenchymal characteristics, such as reduced intracellular adhesion and increased migration 7, 8, 9, 10, 11. In addition, cell proliferation and EMT are always accompanied by the dynamic changes of gene expression. One of the hallmarks to evaluate EMT is the reduction in E‐cadherin expression, which is considered an active suppressor of invasion and growth of many epithelial cancers 8, 9, 10, 11.

MicroRNAs (miRNAs) are a family of small non‐coding RNAs that could bind to the partially complementary recognition sequence of target mRNAs, leading to either the degradation of mRNAs or the inhibition of translation 4, 5, 12, 13, 14, 15. MicroRNAs have been reported to regulate different properties of cancers, such as cancer cell proliferation, migration, invasiveness, EMT, and so on, by repressing their target gene expression 5, 8, 16, 17, 18. Recent evidences indicate that several miRNAs have been involved in the tumorigenic driver pathways in NSCLC, which would be developed as a new therapeutic strategy of NSCLC 2, 15. Therefore, it is of great concern to investigate the roles and potential mechanisms of key miRNAs in tumorigenic driver pathways. MiR‐138 has been proven to play important roles in a number of cancer types and regulate different biological processes 8, 16, 17, 18, 19, 20. Recent studies have shown that miR‐138 was frequently down‐regulated in NSCLC and lung cancer cell lines. Zhang *et al*. and Ye *et al*. showed that miR‐138 could inhibit NSCLC cell growth and tumour growth in nude mice by suppressing the expression of its target genes the enhancer of zeste homolog 2 (EZH2) and 3‐phosphoinositide‐dependent protein kinase‐1 (PDK1) 16, 19. In general, however, one miRNA has numerous target genes, and a miRNA may be multifunctional, which means that miR‐138 may inhibit NSCLC cell growth by targeting some other genes associated with the EMT of NSCLC 5, 7, 17, 21, 22.

To further understand the regulatory mechanisms of miR‐138 in NSCLC progression, we in this study chose NSCLC A549 and 95‐D cells, of which 95‐D cell is a highly metastatic human NSCLC cell line that is suitable for studying some specific properties of NSCLC, such as EMT 5, 7, 23, 24. First, we examined the effect of miR‐138 on the NSCLC cell growth and found that the overexpression of miR‐138 inhibited cell growth and arrested cell cycle at G0/G1 by suppressing the expression of G‐protein‐coupled receptor kinase‐interacting protein 1 (GIT1). Importantly, we unexpectedly found that miR‐138 overexpression induced the reversion of EMT with decreased Slug expression and increased zonula occludens‐1 (ZO‐1) and E‐cad expressions, accompanied by reduced migration and invasion abilities. Furthermore, we demonstrated that both GIT1 and semaphorin 4C (SEMA4C) were direct functional targets of miR‐138 in the progress of NSCLC EMT. Our study not only makes a significant contribution to the understanding of the roles and molecular mechanisms of miR‐138, GIT1 and SEMA4C in NSCLC progression, but also the data may be translated into new therapeutics and/or prognostic biomarkers for NSCLC.

## Materials and methods

### Cell culture

Non‐small‐cell lung cancer cell lines A549 and 95‐D were purchased from the Type Culture Collection of the Chinese Academy of Sciences (Shanghai, China), and cultured in RPMI‐1640 medium (Life Sciences, Corning, NY, USA) supplemented with foetal bovine serum (FBS, Gemini, Woodland, CA, USA) at a final concentration of 10% FBS. All the cells were cultured at 37°C in a humidified incubator with 5% CO_2_.

### Prediction of miR‐138 targets and siRNA design

MiR‐138 target genes were predicted using the TargetScan (http://www.targetscan.org/) and PicTar (http://pictar.mdc-berlin.de/) algorithms. For this study, genes that were predicted by the two methods were defined as potential miR‐138 targets. Small interfering RNAs (siRNAs) against GIT1 and SEMA4C were designed using the BLOCK‐iT RNAi Designer (Life Technologies, Carlsbad, CA, USA) at http://rnaidesigner.lifetechnologies.com/rnaiexpress/and we ordered two or three siRNAs sequences of each gene for the following studies (Table 1).

**Table 1:** Sequences of miRNA mimics and siRNAs

Name	Sequence (5′–3′)
Anti‐miR‐138	CGGCCUGAUUCACAACACCAGCU
hsa‐miR‐138	Sense: AGCUGGUGUUGUGAAUCAGGCCGTT
	Anti‐sense: CGGCCUGAUUCACAACACCAGCUTT
GIT1 siRNA‐1	Sense: CGAGCUGCUUGUAGUGUAUTT
	Anti‐sense: AUACACUACAAGCAGCUCGTT
GIT1 siRNA‐2	Sense: GUGCCAAUAUGAGCUCAGUTT
	Anti‐sense: AGUGAGCUCAUAUUGGCACTT
GIT1 siRNA‐3	Sense: CCUUGAUCAUCGACAUUCUTT
	Anti‐sense: AGAAUGUCGAUGAUCAAGGTT
SEMA4C siRNA‐1	Sense: CCGACAACAUCCUCAACUUTT
	Anti‐sense: AAGUUGAGGAUGUUGUCGGTT
SEMA4C siRNA‐2	Sense: GCAACCUCCGUGGCAGUAATT
	Anti‐sense: UUACUGCCACGGAGGUUGCTT
Control	Sense: UUCUCCGAACGUGUCACGUTT
	Anti‐sense: ACGUGACACGUUCGGAGAATT

### DNA constructs

To construct a luciferase reporter vector, a 716 bp fragment from the 3′UTR of GIT1 (position 2954‐3669, NM_001085454.1) was amplified using cDNA templates transcribed from 95‐D RNA with the following primers (Invitrogen, Life Technologies): WT‐GIT1‐For and WT‐GIT1‐Rev (Table 2). The corresponding mutant constructs were created by the following primer pairs: MU‐GIT1‐1 construct with MU‐GIT1‐For and WT‐GIT1‐Rev, MU‐GIT1‐2 construct with WT‐GIT1‐For and MU‐GIT1‐Rev, and MU‐GIT1 construct with MU‐GIT1‐For and MU‐GIT1‐Rev. In addition, a 200 bp fragment from the 3′UTR of SEMA4C (position 2686‐2885, NM_017789.4) also was amplified using the same cDNA templates with the following primers: WT‐SEMA4C‐For and WT‐SEMA4C‐Rev. The corresponding mutant constructs were created by the following primer pairs: MU‐SEMA4C‐1 construct with MU‐SEMA4C‐For and WT‐SEMA4C‐Rev, MU‐SEMA4C‐2 construct with WT‐SEMA4C‐For and MU‐SEMA4C‐Rev, and MU‐SEMA4C construct with MU‐SEMA4C‐For and MU‐SEMA4C‐Rev. All the wild‐type and mutant 3′UTR were cloned into the downstream of the renilla luciferase gene in psiCHECK‐2 vector (Promega, Madison, WI, USA). Moreover, we constructed the GIT1 and SEMA4C overexpression vectors using the same cDNA templates and specific primers, of which the primers for the open reading frame (ORF) of GIT1 (position 200‐2892, NM_001085454.1) were GIT1‐For and GIT1‐Rev, and the primers for the ORF of SEMA4C (position 62‐3041, NM_017789.4) were SEMA4C‐For and SEMA4C‐Rev. After amplification, the ORF fragments were inserted to the multiple cloning sites of pcDNA 3.1(+) vector (Invitrogen, Life Technologies). All constructs were confirmed by DNA sequencing (Life Technologies).

**Table 2:** Primers used for PCR and qRT‐PCR

Name	Sequence (5′–3′)
GIT1‐For	CTAGCTAGCGCGTCGCCGCTGAGGA
GIT1‐Rev	GGAATTCGGGGCGCATGTACGGA
WT‐GIT1‐For	CCGCTCGAGTAGTCTCCATGGATGTCC
WT‐GIT1‐Rev	CGGGATCCCCAGGGAAGAAGCGGGAT
MU‐GIT1‐For	CCGCTCGAGTAGTCTCCATGGATGTCCCTGCCCTGTAGCGTGGTGCCCCT
MU‐GIT1‐Rev	CGGGATCCCCAGGGAAGAAGCGGGATGGGGAGAGAGCACCACGGTCCC
SEMA4C‐For	CCCAAGCTTGTGGACGGCGGTCAGA
SEMA4C‐Rev	GCTCTAGAGACAGAAACAGGAAGGCTATG
WT‐SEMA4C‐For	CCGCTCGAGGAAGCGTGGGAGGTGTA
WT‐SEMA4C‐Rev	CGGGATCCAGAGAGGAGGAAGATGCC
MU‐SEMA4C‐For	CCGCTCGAGGAAGCGTGGGAGGTGTAGCTCCTACTTTTGCACAGGGTGGTGCTACCT
MU‐SEMA4C‐Rev	CGGGATCCAGAGAGGAGGAAGATGCCTTCTGCGAGGCACCACCCCTG
miR‐138 For	CTGGAGAGCTGGTGTTGTGAAT
U6 For	CTCGCTTCGGCAGCACA
U6 Rev	AACGCTTCACGAATTTGCGT
GIT1 QFor	CGACGACCAACACGACTACGA
GIT1 QRev	CACCTTTGCCTCCGATGTAG
SEMA4C QFor	CAGCCCTACAATGCCTCCC
SEMA4C QRev	GCCGAGTACAGCTCACCAT

### Cell transfection

MicroRNA mimics or siRNA (GenePharma, Shanghai, China) at a final concentration 50 nM, with or without DNA plasmids at a final concentration 900 ng/μl were transfected into A549 or 95‐D cells with X‐tremeGENE siRNA Transfection Reagent (Roche, Indianapolis, IN, USA) according to the manufacturer's instructions. Forty‐eight hours after transfection, cells were analysed as required.

### Cell proliferation assay

At 24 hrs before transfection, cells were seeded into 24‐well plates at a density of 2 × 10^4^ cells/well. After transfection, cell proliferation was measured by the methyl thiazolyl tetrazolium (MTT) assay at 48 hrs. Briefly, 0.4 ml of MTT solution (5 mg/ml in PBS) was added to each well and cells were incubated for 4 hrs at 37°C, then the supernatant medium was replaced by 0.2 ml of dimethyl sulfoxide. After 10 min., the optical density was measured at 490 nm with a SpectraMax L spectrophotometer.

### Cell‐cycle analysis

Cells (1 × 10^6^) were harvested at 48 hrs after transfection, washed once with PBS and fixed in 70% ethanol (diluted with PBS) at 4°C overnight. Then the cells were harvested, washed once with PBS again and incubated with 0.5 ml staining fluid containing 50 μg/ml propidium iodide, 0.1 mg/ml ribonuclease A and 0.2% Triton X‐100 for 30 min. at 4°C. Cell‐cycle distribution was measured by BD Accuri C6 flow cytometry (BD Biosciences, San Jose, CA, USA) and the data were analysed by ModFit LT Version 4.1 Software (Verity Software House, Topsham, ME, USA).

### Immunofluorescent staining

Cells were fixed in 4% paraformaldehyde for 30 min. and washed with pre‐cooling PBS for three times, 5 min. each time. Then, fixed cells were blocked with 1% bovine serum albumin (AMRESCO, Rudner, PA,USA) and 0.25% Triton X‐100 (MP Biomedicals, Santa Ana, CA, USA) in PBS for 1 hr at room temperature before incubated with primary antibody Anti‐Ki67 (1:300, cat. no. ab92742; Abcam, Cambridge, MA, USA) overnight at 4°C. After PBS rinses, cells were incubated with secondary antibodies conjugated with Alexa Fluor 488 (1:1000, cat. no. ab150077; Abcam) for 1 hr at room temperature. The cell nucleus was detected by 4‘,6‐Diamidine‐2‘‐phenylindole dihydrochloriden (DAPI, cat. no. ab104139; Abcam). Images were acquired using a fluorescent microscope (Olympus, Tokyo, Japan). Ki67‐positive cells were counted and normalized to total DAPI‐positive cell numbers.

### Wound healing assay

Cells were seeded into 12‐well plates and cultured in complete medium containing 10% FBS so that the cells would reach confluence at 24 hrs after transfection. Then, a straight scratch wound was made on the cell monolayers by a pipette. Next, the complete medium was replaced with medium containing 4% FBS and the wound area was observed and measured by microscope over a 24 hrs period. The same visual field was marked and used throughout the assay. Healing distance between the wound (%) = [Gap distance (*T*_0_ − *T*_24_)/Gap distance *T*_0_] × 100% (where *T*_0_ is the Gap distance when the wound was made initially at 0 hr, and *T*_24_ is the Gap distance after the wound was healing for 24 hrs). Three independent experiments were performed.

### Invasion assay

A total of 2 × 10^5^ cells/well, suspended in serum free medium, were seeded into the upper chambers with 8.0 μm PET membrane inserts (Corning Costar, Tewksbury, MA, USA), which have been coated with 30% of BD Matrigel matrix (BD Biosciences). The bottom chambers were soaked by complete medium. After 48 hrs incubation, cells remaining in the upper chamber were removed. Cells adhering to the lower membrane were fixed with methanol and stained with crystal violet before counted. Three independent experiments were performed.

### Dual luciferase reporter assay

95‐D cells were plated into 24‐well plates at 24 hrs before transfection and the cells would reach a confluence of 90% when transfected. psiCHECK‐2 containing the wild‐type or mutated potential target gene 3′UTRs and mRNA were cotransfected into 95‐D cells by X‐tremeGENE siRNA Transfection Reagent. Relative Renilla luciferase activity normalized to firefly luciferase activity was measured at 48 hrs after transfection using the Dual‐Glo^®^ Luciferase Assay System (Promega) on a SpectraMax L spectrophotometer (Molecular Devices, Sunnyvale,CA, USA).

### Quantitative real‐time PCR analysis

The relative expression level of miR‐138 (normalized to U6) was determined using All‐in‐One^™^ miRNA quantitative real‐time PCR (qRT‐PCR) Detection Kit (GeneCopoeia, Rockville, MD, USA) according to the manufacturer's protocol in a Bio‐Rad CFX96 Real‐Time PCR Detection System (Bio‐Rad, Hercules, CA, USA). However, the relative expression levels of GIT1 and SEMA4C (normalized to β‐actin) were determined using FastStart Universal SYBR Green Master (Roche). Specific primer sets for miR‐138 (miR‐138 For), U6 (U6 For/Rev), GIT1 (GIT1 QFor/QRev) and SEMA4C (SEMA4C QFor/QRev) were obtained from Invitrogen (Table 2). The relative expression levels of the miRNA and mRNAs were determined using the 2^−∆∆Ct^ analysis method, where U6 was used as an internal reference for miR‐138 and β‐actin for mRNAs. All reactions were performed in triplicate.

### Western blotting

Cells were harvested and lysed with 0.5% NP‐40 cell lysis buffer [50 mM Tris‐Cl, 15 mM NaCl, 5 mM ethylenediaminetetraacetic acid, 0.5% NP‐40, 1 mM Phenylmethanesulfonyl fluoride (PMSF), pH 6.8] for 20 min. on ice. Lysates were then centrifuged at 12,000 × g at 4°C for 10 min. and the supernatants were harvested for research. Proteins were separated on 10% SDS‐PAGE gels and then transferred onto polyvinylidene difluoride membranes. Primary antibodies against GIT1 (cat. no. 2919; CST, Danvers, MA, USA), SEMA4C (cat. no. sc‐136445; Santa Cruz Biotechnology, Santa Cruz, CA, USA), β‐actin (cat. no. SC47778; Santa Cruz Biotechnology) or antibodies from EMT Antibody Sampler Kit (cat. no. 9782; CST) were incubated with the membranes overnight at 4°C (1:1000 dilution) respectively. The membranes were washed and incubated with respective secondary antibodies and were visualized by SignalFire^™^ ECL Reagent (CST) according to the manufacturer's instructions in a Bio‐Rad Molecular Imager ChemiDoc XRS (Bio‐Rad). Shown are the representative data from three individual experiments.

### Statistical analysis

Data are presented as means ± S.D. of three independent experiments. Differences between groups were analysed by GraphPad Prism 6 software (GraphPad Software, La Jolla, CA, USA) with Student's *t*‐test. Differences were considered statistically significant at *P* < 0.05, *P < 0.05, **P < 0.01, and ***P < 0.001.

## Results

### Overexpression of miR‐138 suppresses the proliferation of NSCLC cells

**Figure 1:**
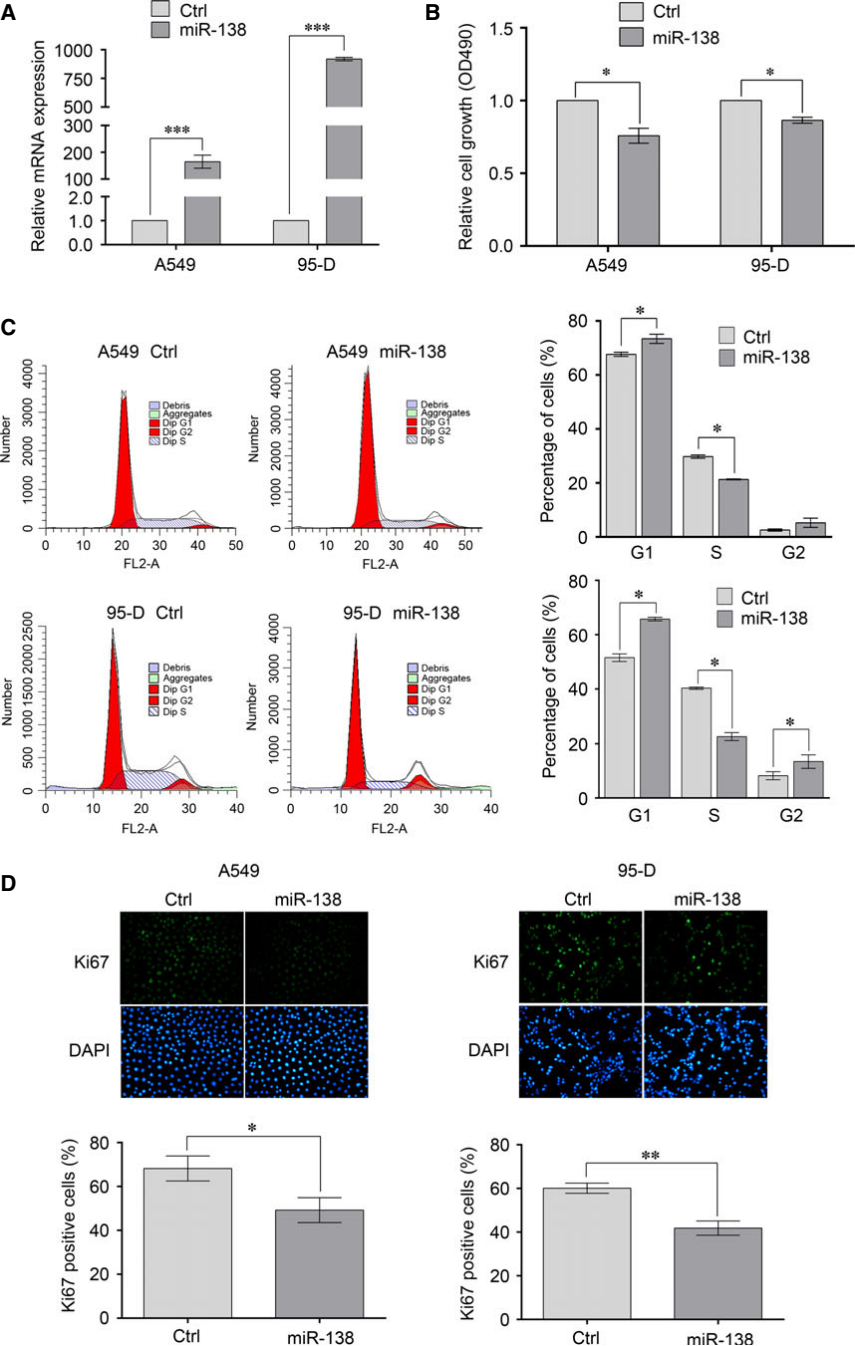
Overexpression of miR‐138 suppresses the proliferation of non‐small‐cell lung cancer (NSCLC) cells. A549 and 95‐D cells were transfected with miR‐138 mimics or negative control mimics. (**A**) 48 hrs after transfection, RNA was extracted and the level of miR‐138 was determined by qRT‐PCR analysis. The amount of miR‐138 was normalized to U6. (**B**) 48 hrs after transfection, cell proliferation was evaluated by methyl thiazolyl tetrazolium (MTT) assay. (**C**) 48 hrs after transfection, cell cycle was evaluated using propidium iodide staining. (**D**) 48 hrs after transfection, cells proliferation was evaluated by Ki67 immunostaining assay. Results are presentative of three independent experiments and the error bars refer to S.D. *P < 0.05 and **P < 0.01.

To explore the role of miR‐138 in NSCLC cells, we first investigated the effects of miR‐138 overexpression on the proliferation and cell cycle of A549 and 95‐D cells. Increased level of miR‐138 was confirmed by qRT‐PCR (Fig. 1A). As shown in Figure 1B, ectopic transfection of the miR‐138 mimic into A549 and 95‐D cells obviously suppressed the cell growth, examined by the MTT assay. Consistent with the results, miR‐138 overexpression increased the proportion of cells in G0/G1 phase, and decreased the cells in S phase (Fig. 1C). To gain deep insight, we further examined the effects of miR‐138 on the proliferation of A549 and 95‐D cells by Ki67 immunostaining analysis. Compared with negative control, the ratio of Ki67‐positive cells was reduced when miR‐138 was overexpressed (Fig. 1D). Taken together, our results indicate that miR‐138 suppressed the growth by inducing cell‐cycle arrest at G0/G1 phase in NSCLC cells.

### Overexpression of miR‐138 reverses the EMT of NSCLC cells

**Figure 2:**
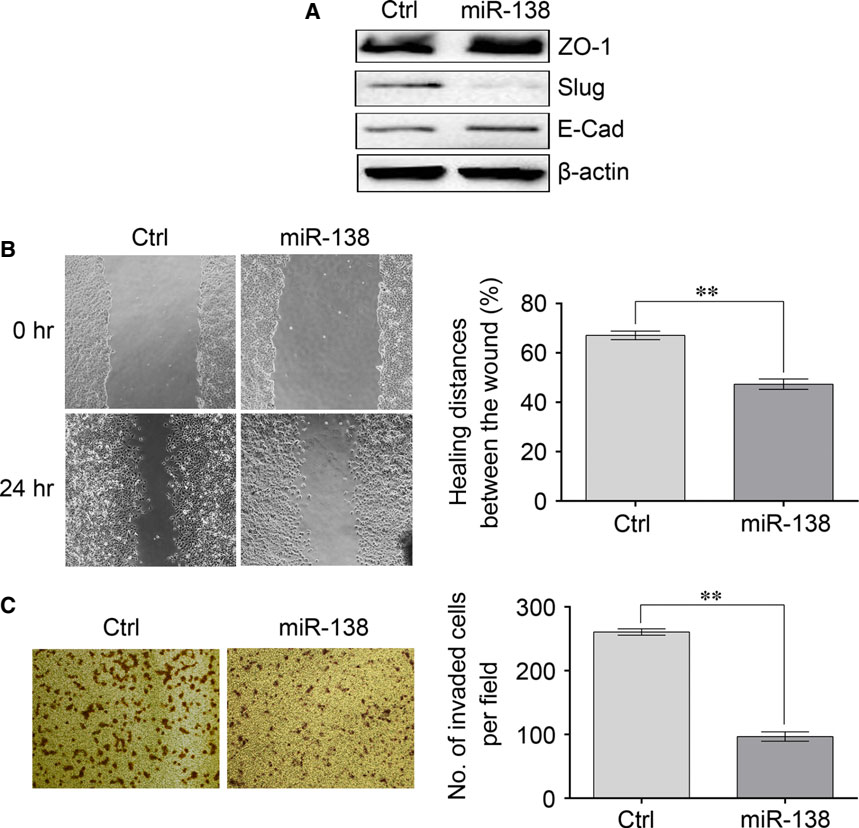
Overexpression of miR‐138 reverses the epithelial‐mesenchymal transition (EMT) of non‐small‐cell lung cancer (NSCLC) cells. 95‐D cells were transfected with miR‐138 mimics or negative control mimics. (**A**) 48 hrs after transfection, the total proteins were obtained from lysed cells and the expressions of ZO‐1, E‐Cad and Slug were determined by Western blotting. β‐actin was used as a loading control. (**B**) 24 hrs after transfection, cell migration was evaluated by wound healing assay. (**C**) 24 hrs after transfection, cell invasion was evaluated using Matrigel‐coated Transwell assay. Results are presentative of three independent experiments and the error bars refer to S.D. **P < 0.01.

We next determined whether the exogenous expression of miR‐138 could affect properties of NSCLC cells, such as EMT. We chose 95‐D cell, a highly metastatic human NSCLC cell, for the following study. As shown in Figure 2A, miR‐138 overexpression led to a significant decrease in Slug expression and an increase in ZO‐1 and E‐cad expressions, which are the hallmarks of EMT reversion. Epithelial‐mesenchymal transition is always associated with some malignant properties, such as migration and invasion. Therefore, we further examined the effects of miR‐138 on the migration and invasion of NSCLC cells by wound healing and Matrigel‐coated Transwell assays. As illustrated in Figure 2B and C, the increased miR‐138 level in 95‐D cells resulted in reduced cell migration and invasion, which is in agreement with the coordinated relationship between EMT and cell motility and invasion. In conclusion, our results showed that miR‐138 reversed EMT by decreasing Slug expression and increasing ZO‐1 and E‐cad expressions in NSCLC cells.

### GIT1 and SEMA4C are direct targets of miR‐138

**Figure 3:**
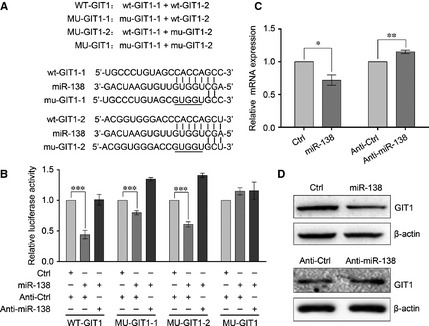
Identification of GIT1 as a target of miR‐138. (**A**) Diagram of the luciferase reporter constructs. Two predicted miR‐138‐targeting sequences were located in the 3′‐UTR of the GIT1 mRNA (wt‐GIT1‐1, wt‐GIT1‐2). Mutation was introduced into the target sites (underlined nucleotides) for generating mutated reporter constructs. (**B**) Dual luciferase reporter assay. Luciferase reporter constructs that included wide‐type or mutant GIT1 3′UTR and miR‐138 mimics or inhibitor or negative control mimics were cotransfected into 95‐D cells. 48 hrs after co‐transfection, luciferase activity was determined. (**C**) The expression of GIT1 mRNA in 95‐D cells transfected with miR‐138 mimics or Anti‐miR‐138 was examined by qRT‐PCR analysis, normalized to β‐actin. (**D**) The expression of GIT1 protein in 95‐D cells transfected with miR‐138 mimics or Anti‐miR‐138 was determined by Western blotting. β‐actin was used as an internal control. Results are presentative of three independent experiments and the error bars refer to S.D. *P < 0.05, **P < 0.01, and ***P < 0.001.

To further understand the molecular mechanism of miR‐138‐induced growth inhibition and EMT reversion in NSCLC cells, we adopted bioinformatic algorithms (TargetScan and PicTar) to predict a large number of potential miR‐138 target genes. Among them, GIT1 and SEMA4C were found to have putative miR‐138 binding sites in their 3′UTRs. To verify whether GIT1 is a direct target of miR‐138, a 716‐bp fragment within the 3′UTR of GIT1 that contains two putative miR‐138 binding sites was cloned into psiCHECK‐2 vector at the downstream of the renilla luciferase gene (WT‐GIT1). Three control reporter vectors were also developed in which one or two of seed regions of the miR‐138 binding sites were mutated (MU‐GIT1‐1, MU‐GIT1‐2 and MU‐GIT1) (Fig. 3A). Then, 95‐D cells were cotransfected with plasmids and RNA as illustrated in Figure 3B. The reporter assay showed that miR‐138 overexpression decreased the luciferase reporter activities of WT‐GIT1, MU‐GIT1‐1 and MU‐GIT1‐2, and this inhibition was significantly reduced by co‐transfection with the Anti‐miR‐138, indicating that miR‐138 could bind to the two putative binding sites in GIT1 3′UTR. Mutation of both the two miR‐138 binding sites abolished the inhibition by miR‐138 (Fig. 3B). In addition, qRT‐PCR and Western blot analyses showed that miR‐138 overexpression significantly decreased the levels of GIT1 mRNA and protein expression in 95‐D cells, and Anti‐miR‐138 overexpression increased the levels of GIT1 mRNA and protein expression (Fig. 3C and D). These results indicate that GIT1 is a direct target of miR‐138 in NSCLC cells.

**Figure 4:**
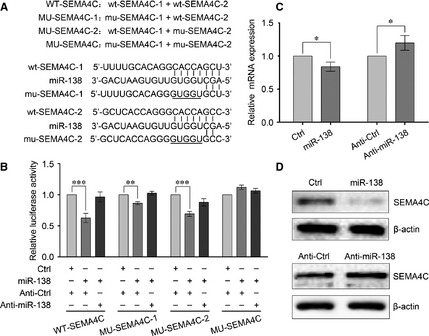
Identification of SEMA4C as a target of miR‐138. (**A**) Diagram of the luciferase reporter constructs. Two predicted miR‐138‐targeting sequences were located in the 3′‐UTR of the SEMA4C mRNA (wt‐SEMA4C‐1, wt‐SEMA4C‐2). Mutation was introduced into the target sites (underlined nucleotides) for generating mutated reporter constructs. (**B**) Dual luciferase reporter assay. Luciferase activity was assayed using the methods just like that of Figure 3. (**C**) The expression of SEMA4C mRNA was examined by qRT‐PCR analysis, normalized to β‐actin. (**D**) The expression of SEMA4C protein was probed by Western blotting. β‐actin was used as an internal control. Results are presentative of three independent experiments and the error bars refer to S.D. *P < 0.05, **P < 0.01 and ***P < 0.001.

Secondly, to validate whether SEMA4C is also a direct target of miR‐138, a 200‐bp fragment within the 3′UTR of SEMA4C that also contains two putative miR‐138 binding sites was cloned into psiCHECK‐2 vector (WT‐SEMA4C) and three control reporter vectors were also constructed and named MU‐SEMA4C‐1, MU‐SEMA4C‐2, and MU‐SEMA4C (Fig. 4A). As shown in Figure 4B, miR‐138 overexpression decreased the luciferase reporter activities of WT‐SEMA4C, MU‐SEMA4C‐1 and MU‐SEMA4C‐2 and this inhibition could be significantly reduced by co‐transfection with the Anti‐miR‐138, whereas no significant difference was detected with MU‐SEMA4C, indicating that miR‐138 could bind to the two putative binding sites in SEMA4C 3′UTR as well. Additionally, qRT‐PCR and Western blotting showed that miR‐138 overexpression significantly decreased the levels of SEMA4C mRNA and protein expression in 95‐D cells, and Anti‐miR‐138 overexpression increased the levels of SEMA4C mRNA and protein expression (Fig. 4C and D). Taken together, we speculate that SEMA4C is another direct target of miR‐138 too.

### MiR‐138 inhibits the proliferation of NSCLC cells by targeting GIT1

**Figure 5:**
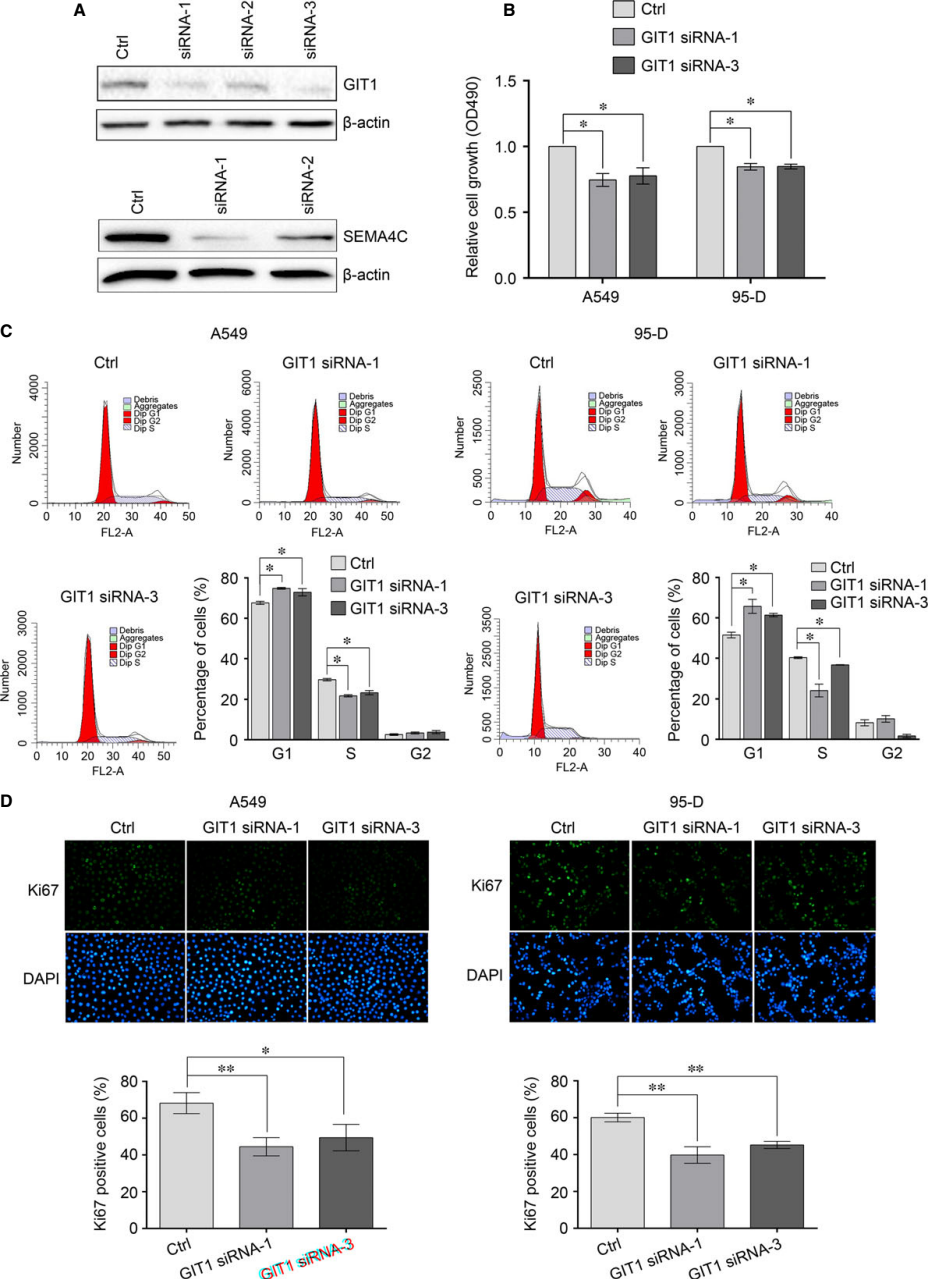
Knockdown of GIT1 inhibits the proliferation of non‐small‐cell lung cancer (NSCLC) cells. (**A**) Silencing of GIT1 and SEMA4C by siRNAs was determined by Western blotting. 95‐D cells were transfected with three GIT1 siRNAs or two SEMA4C siRNAsrespectively. 48 hrs after transfection, the total proteins were extracted and the expressions of GIT1 and SEMA4C were determined by Western blotting. β‐actin was used as an internal control. (**B**) Cells proliferation was evaluated by methyl thiazolyl tetrazolium (MTT) assay. (**C**) Cell cycle was evaluated using propidium iodide staining and flow cytometry. (**D**) Cells proliferation was evaluated by Ki67 immunostaining assay. Results are presentative of three independent experiments and the error bars refer to S.D. *P < 0.05 and **P < 0.01.

To assess whether miR‐138 inhibits the proliferation of NSCLC cells by targeting GIT1 or SEMA4C, we first asked whether the knockdown of GIT1 or SEMA4C can impair the proliferation of NSCLC cells. Three siRNAs against GIT1 and two siRNAs against SEMA4C were designed (Table 1). As shown in Figure 5A, the transfection of GIT1 siRNA‐1 and siRNA‐3 and SEMA4C siRNA‐1 and siRNA‐2 caused significant reduction in the protein levels of GIT1 and SEMA4C respectively in 95‐D cells, which would be used for the following studies. Then we examined the effects of GIT1 siRNA and SEMA4C siRNA on the proliferation and cell‐cycle distribution of NSCLC cells. As illustrated in Figure 5B and C, the reduction in GIT1 expression significantly decreased cell proliferation and arrested cell cycle at G0/G1 phase, which was similar to the results obtained by miR‐138 overexpression. The inhibitional effect was further confirmed by Ki67 immunostaining analysis (Fig. 5D). However, we found that the silencing of SEMA4C expression did not impair the proliferation of NSCLC cells (Fig. S1).

**Figure 6:**
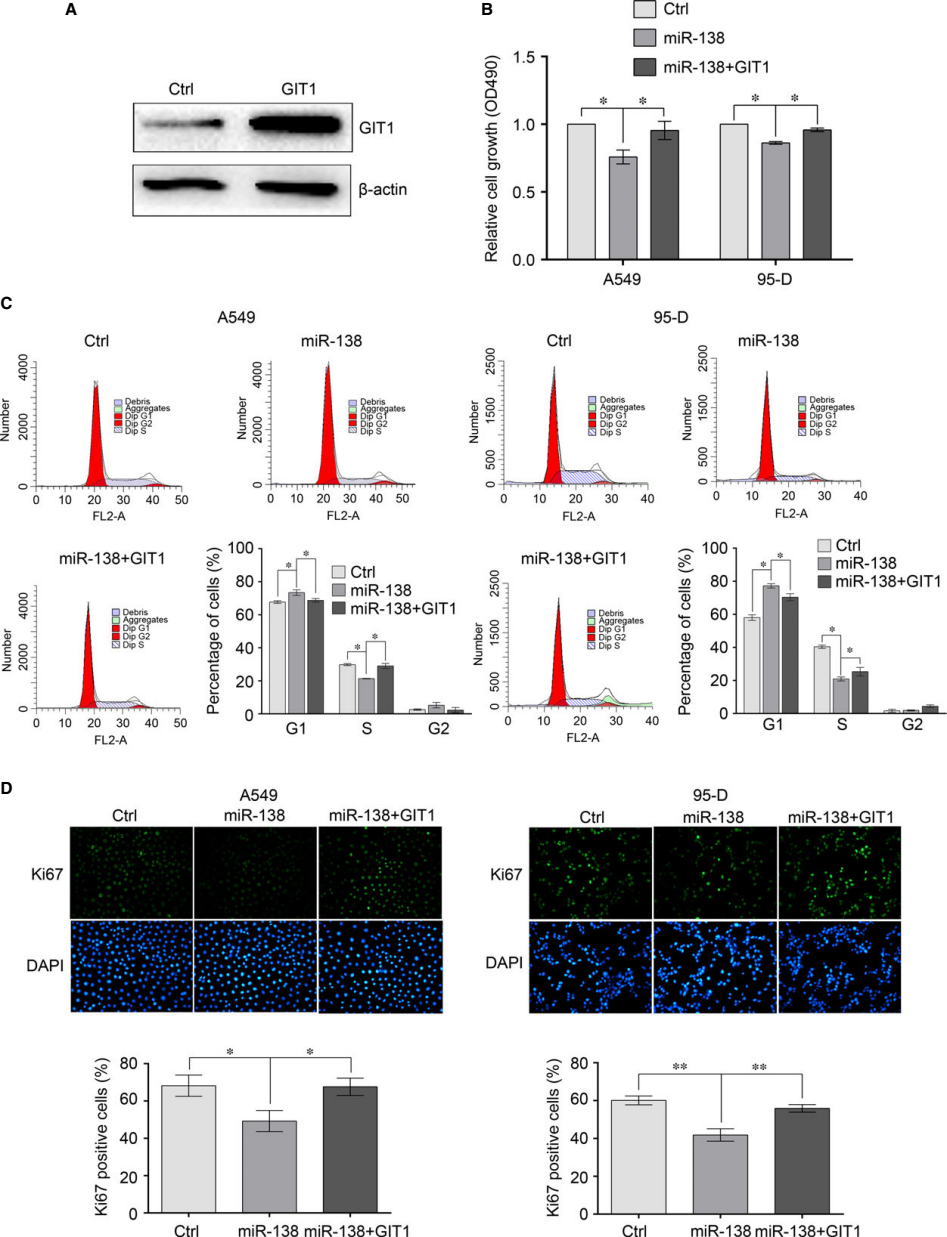
Ectopic expression of GIT1 rescues miR‐138‐induced cell growth inhibition. (**A**) Overexpression of GIT1 was determined by Western blot analysis. 95‐D cells were transfected with the GIT1 overexpression plasmids. 48 hrs after transfection, the total proteins were extracted and the expression of GIT1 was determined by Western blotting. β‐actin was used as an internal control. (**B**) Cells proliferation was evaluated by methyl thiazolyl tetrazolium (MTT) assay. A549 and 95‐D cells were transfected with miR‐138 mimics or control mimics, or co‐transfected with miR‐138 mimics and GIT1 overexpression plasmids. 48 hrs after transfection, cells proliferation was examined. (**C**) Cell cycle was evaluated using propidium iodide staining and flow cytometry. (**D**) Cells proliferation was evaluated by Ki67 immunostaining assay. Results are presentative of three independent experiments and the error bars refer to S.D. *P < 0.05 and **P < 0.01.

Subsequently, we evaluated whether exogenous overexpression of GIT1 could rescue the suppressive effect of miR‐138 on NSCLC cells. A549 and 95‐D cells were co‐infected with miR‐138 mimics and recombinant plasmids only containing GIT1 ORF. The overexpression of GIT1 was determined by Western blotting (Fig. 6A). Then cell proliferation and cell cycle were examined and the data showed that the forced expression of GIT1 significantly rescued miR‐138‐induced cell growth inhibition and cell‐cycle arrest (Fig. 6B–D). Taken together, these results indicate that miR‐138 inhibits the proliferation of NSCLC cells partly by down‐regulating GIT1.

### MiR‐138 reverses EMT of NSCLC cells by targeting SEMA4C and GIT1

**Figure 7:**
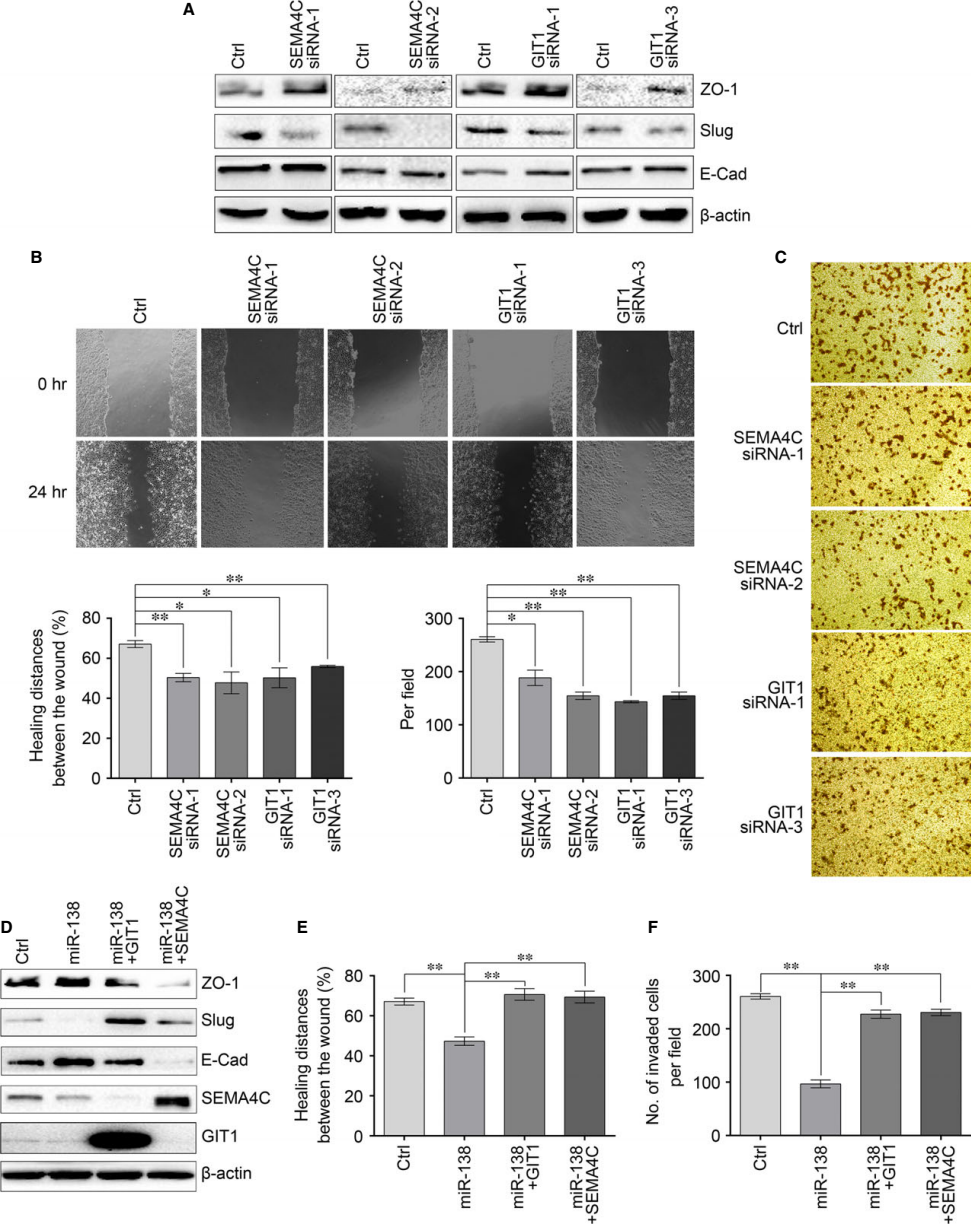
MiR‐138 regulates epithelial‐mesenchymal transition (EMT) by targeting GIT1 and SEMA4C. (**A**) The expressions of ZO‐1, E‐Cad and Slug were determined by Western blotting after the knockdown of SEMA4C or GIT1 in 95‐D cells. β‐actin was used as a loading control. (**B**) 24 hrs after knockdown of SEMA4C or GIT1 in 95‐D cells, cell migration was evaluated by wound healing assay. (**C**) 24 hrs after knockdown of SEMA4C or GIT1 in 95‐D cells, cell invasion was evaluated using Matrigel‐coated Transwell assay. (**D**) The expressions of ZO‐1, E‐Cad and Slug were determined by Western blot analysis. 95‐D cells were transfected with miR‐138 mimics or control mimics, or cotransfected with miR‐138 mimics and GIT1 or SEMA4C overexpression plasmids. Forty‐eight hours after transfection, the expressions of ZO‐1, E‐Cad and Slug were determined by Western blotting. β‐actin was used as an internal control. (**E**) 24 hrs after transfection, cell migration was evaluated by wound healing assay. (**F**) 24 hrs after transfection, cell invasion was evaluated using Matrigel‐coated Transwell assay. Results are presentative of three independent experiments and the error bars refer to S.D. *P < 0.05 and **P < 0.01.

To determine whether SEMA4C or GIT1 is a critical mediator of miR‐138′ role on EMT of NSCLC cells, we firstly tested the effect of SEMA4C or GIT1 knockdown on the EMT of NSCLC cells. As illustrated in Figure 7A, the knockdown of SEMA4C or GIT1 expression led to a decrease in Slug expression and an increase in ZO‐1 and E‐cad expressions, similar to those induced by miR‐138 overexpression. And likewise, the cell migration and invasion abilities were also suppressed by the knockdown of SEMA4C or GIT1 (Fig. 7B and C). Next, we examined whether the overexpression of SEMA4C or GIT1 could rescue the suppressive effect of miR‐138 overexpression. As shown in Figure 7D–F, as compared with the miR‐138, the forced expression of SEMA4C or GIT1 not only significantly decreased the expressions of ZO‐1 and E‐cad, and increased the expression of Slug; but also increased the cell migration and invasion abilities. Taken together, these results indicate that miR‐138 reverses EMT of NSCLC cells, at least in part, by targeting SEMA4C and GIT1.

## Discussion

MicroRNA is a kind of endogenous small non‐coding RNAs that could participate in many biological processes, including cell proliferation, apoptosis, cell differentiation and development 9, 16, 17, 25. It is believed that the dysregulation of miRNAs is closely related to the development and progression of a number of cancers 4, 5, 12, 13, 21. The down‐regulation of miR‐138 in NSCLC has been consistently observed by multiple laboratories 16, 19. In this study, we determined that the exogenous expression of miR‐138 in A549 and 95‐D cells inhibited the cell growth and arrested cell cycle at G0/G1 by suppressing the expression of GIT1. In addition, we even found that the overexpression of miR‐138 could reverse the EMT by inhibiting the expressions of GIT1 and SEMA4C.

Cell proliferation is a complicated progress, which is always regulated by a series of genes 16, 19, 23, 26. To further understand the molecular mechanism of miR‐138‐induced growth inhibition in NSCLC cells, we predicted and verified a series of candidate miR‐138 target genes using bioinformatics tools and dual luciferase reporter assay as previously 17, 18, 20, 25. Our results revealed that GIT1 was down‐regulated by the miR‐138 overexpression. GIT1 is a multifunctional protein that has been known to have a central role in cell migration/invasion, focal adhesion, lamellipodia formation, cell growth and so on 26, 27, 28, 29, 30, 31. In cells with high metastasis, GIT1 depletion has been found to reduce α5β1‐integrin‐mediated cell adhesion to fibronectin and collagen, and further inhibit cell migration/invasion and metastasis, with enhanced degradation of paxillin, α5β1 integrin, phospho‐paxillin, phospho‐FAK, EGF/EGFR‐mediated extracellular signal‐regulated kinase (ERK1/2) activation, and matrix metalloproteinases 2 and 9 (MMP2/9) levels and activities 26, 28, 30, 31. Recently, Peng *et al*. have found that GIT1 could interact with methionine adenosyltransferase 2B variants and form a scaffold, which would recruit and activate mitogen/extracellular signal/regulated kinase (MEK) and ERK to promote cell growth and tumorigenesis 26. To reveal the role of GIT1 in NSCLC, we silenced GIT1 with specific siRNA in NSCLC cells, and found that GIT1 knockdown inhibited the cell growth and arrested cell cycle at G0/G1, just like the effect of miR‐138 overexpression on NSCLC cells, whereas ectopic expression of GIT1 partly rescued the suppressive effect of miR‐138. Altogether, we conclude that miR‐138 can modulate NSCLC cell growth by suppressing the expression of GIT1.

Non‐small‐cell lung cancer is not only a disease with malignant proliferation but also with high invasiveness and metastasis 3, 4, 5, 7, 23. MiR‐138 has been reported to regulate a number of essential biological progresses 8, 16, 17, 18, 19, 20. However, the roles of miR‐138 in NSCLC progression have not been completely understood. Therefore, we next examined the effects of miR‐138 on NSCLC cells from the other aspects except for proliferation, such as EMT. Interestingly, we found that the overexpression of miR‐138 could reverse EMT by decreasing Slug expression and increasing ZO‐1 and E‐cad expressions, with decreased migration and invasion abilities. Then we predicted and tested a series of candidate EMT‐related miR‐138 target genes using the same methods as above and found that SEMA4C was also down‐regulated by the miR‐138. SEMA4C has been known to regulate immune cell interactions, angiogenesis, nervous system development, and tumour progression 9, 11, 32, 33. It has been reported that SEMA4C knockdown inhibited phosphorylation of p38 MAPK and reversed TGF‐β1‐induced EMT in renal tubular epithelial cells 11, 33. However, the role of SEMA4C in NSCLC has never been studied yet. In the present study, we silenced SEMA4C by specific siRNA in NSCLC cells and found that SEMA4C knockdown worked just like the miR‐138 overexpression in NSCLC cells, and the ectopic expression of SEMA4C accordingly rescued the reversion effect of miR‐138 on EMT. In addition, GIT1, as a direct target gene of miR‐138, has been shown to have a central role in cell migration/invasion, metastasis and EMT 27, 28, 30. So we also tested the role of GIT1 in NSCLC EMT and found that GIT1 functioned as SEMA4C did, which indicated that GIT1 may be a critical protein modulating invasion and metastasis in the progress of many cancers, such as oral squamous cell carcinoma, breast cancer and NSCLC 26, 27, 30, 31. Therefore, these results indicated that miR‐138 reversed the NSCLC EMT by directly targeting SEMA4C and GIT1.

In summary, the results of our study demonstrated that miR‐138 could regulate NSCLC cell growth by suppressing the expression of GIT1. More importantly, we found that miR‐138 also played major roles in NSCLC EMT by directly targeting SEMA4C and GIT1, which suggests that miR‐138 might be a multifunctional regulator in NSCLC. Taken together, our findings suggest that miR‐138, GIT1 and SEMA4C could be novel and good targets for the development of not only anti‐proliferation, but also anti‐EMT strategies in the treatment of NSCLC. Our results may be translated into valuable biomarkers for NSCLC prognosis, which remains to be validated using a batch of clinical specimens from both NSCLC patients and controls in the future.

## Conflicts of interest

The authors declare that they have no conflict of interest.

## Supporting information


**Figure S1** The effect of SEMA4C knockdown on the proliferation of NSCLC cells.
