# Supplementary figures and images for: MiR‐138 inhibits cell proliferation and reverses epithelial‐mesenchymal transition in non‐small cell lung cancer cells by targeting GIT1 and SEMA4C
Source: J Cell Mol Med. 2015 Aug 18;19(12):2793–805. doi: 10.1111/jcmm.12666 (PMC4687704; doi:10.1111/jcmm.12666)

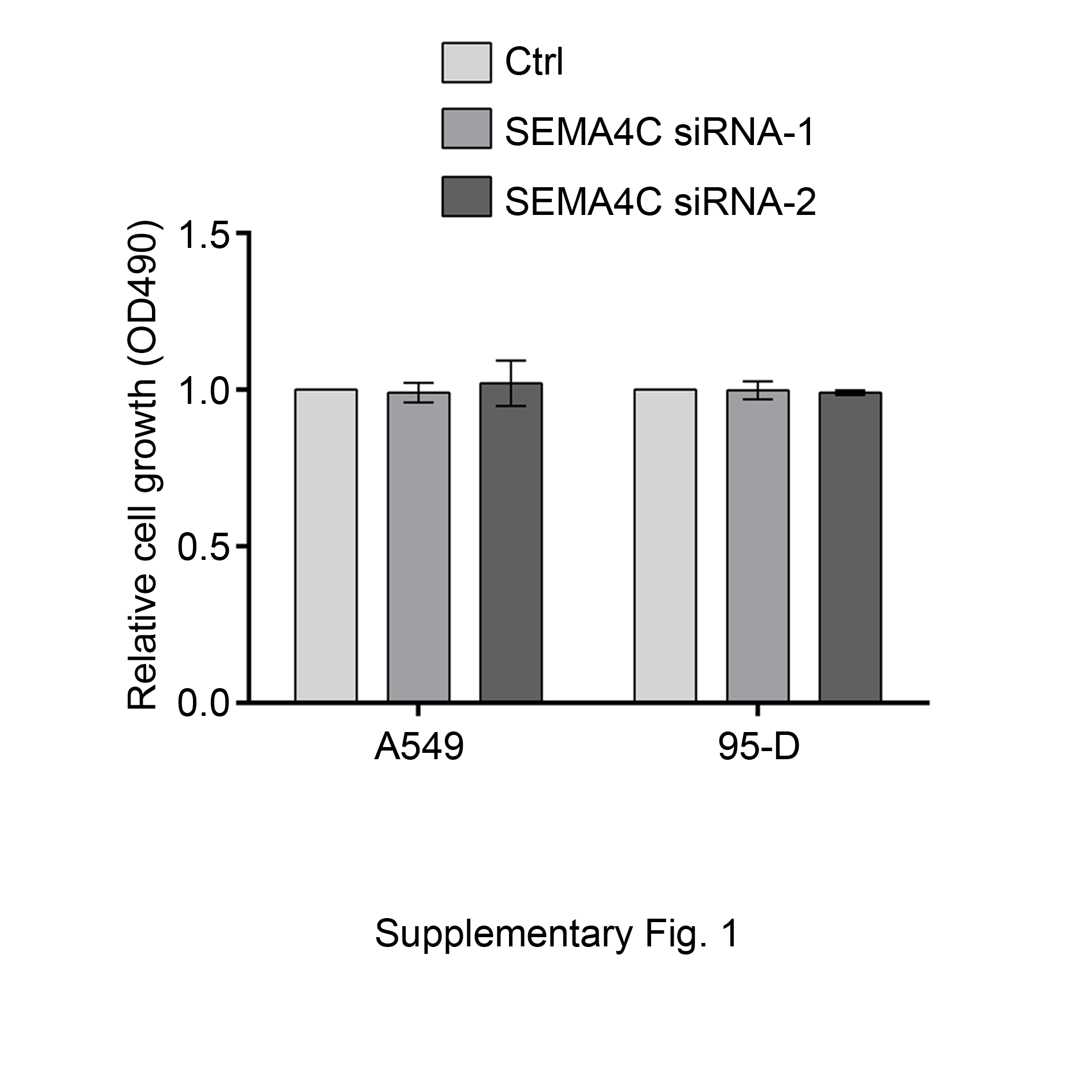

Supplement: Supplementary file 1 — Figure S1 The effect of SEMA4C knockdown on the proliferation of NSCLC cells. [file JCMM-19-2793-s001.tif]
